# Deep tillage combined with biofertilizer following soil fumigation improved chrysanthemum growth by regulating the soil microbiome

**DOI:** 10.1002/mbo3.1045

**Published:** 2020-04-23

**Authors:** Huijie Chen, Shuang Zhao, Jiamiao Zhao, Kaikai Zhang, Jing Jiang, Zhiyong Guan, Sumei Chen, Fadi Chen, Weimin Fang

**Affiliations:** ^1^ State Key Laboratory of Crop Genetics and Germplasm Enhancement Key Laboratory of Landscaping College of Horticulture Ministry of Agriculture and Rural Affairs Nanjing Agricultural University Nanjing China

**Keywords:** biofertilizer, Illumina Hiseq, microbial diversity, monoculture, soil microbiome

## Abstract

Sustained monoculture often leads to the inhibition of plant growth, the decrease of the soil microbial diversity, and changes in soil microbial community composition, particularly to the accumulation of soil‐borne pathogens. In this study, we conducted field experiments to investigate the practical effects of tilling the soil down to a depth of 40 cm (40dp) in combination with dazomet (D) soil fumigation and/or the application of a bio‐organic fertilizer (B) on chrysanthemum growth, with a focus on the potential mechanisms underlying the responses of the soil microbiome. The growth indices of chrysanthemum were significantly (*p* < .05) increased in the DB + 40dp treatment compared to that in other treatments. The weighted and unweighted UniFrac distances in the principal coordinate analysis (PCoA) revealed that soil bacterial and fungal community compositions were separated according to the treatments. The abundance of genera potentially expressing growth promotion, such as *Pseudomonas* and *Bacillus,* was increased in the DB + 40dp treatment. In addition, the combined DB + 40dp treatment enhanced the activities of catalase, urease, sucrase, and β‐d‐glucosidase, and significantly increased the levels of available nitrogen, phosphorus, and potassium in the soil. The redundancy analysis (RDA) implied that the composition of the microbiome was correlated to soil enzymatic activities and soil potassium availability in the rhizosphere soil of chrysanthemum plants. Our findings suggest that the DB + 40dp treatment is a better strategy for improving chrysanthemum growth and regulating the rhizosphere microbiome in monoculture soils than the methods presently employed by commercial chrysanthemum producers.

## Graphical abstract

Tilling the soil down to a depth of 40 cm in combination with Dazomet soil fumigation and the application of biofertilizer promoted chrysanthemum growth, provided substantial control over Fusarium wilt disease, and altered the composition of the rhizosphere soil microbiome, through enhancing the availability of plant nutrients and promoting the presence of plant growth‐promoting rhizomicrobes. The redundancy analysis implied that the composition of the microbiome was correlated to soil enzymatic activities and soil potassium availability in the rhizosphere soil.

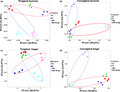

## 1 INTRODUCTION

Chrysanthemum (*Chrysanthemum morifolium* Ramat*.*) is an important ornamental plant, ranked as one of the top four in the world for cut flowers (Dong et al., 2018). Increasing demand from consumers for both cut flowers and potted plants has led to a greater‐than‐ever reliance on monoculture‐based production systems (Zhao, Chen, et al., 2016; Zhao, Tian, et al., 2016). Soil nutrients usually decline with continuous cropping years (Zhang et al., 2018), which, in turn, results in declines in chrysanthemum growth and more frequent incidences of Fusarium wilt disease (FWD). The FWD is soil‐borne wilt infected by *Fusarium oxysporum* f. sp. *chrysanthemi*, which causes a significant decrease in the yield and quality of chrysanthemum (Zhao, Chen, et al., 2016; Zhao, Tian, et al., 2016). Measures such as crop rotation (Yang et al., 2017), screening for effective genetically resistant cultivars (Shukla & Haseeb, 2000), grafting onto resistant rootstocks (Nisini et al., 2002), and agrochemical products (Li, Li, et al., 2017; Li, Tao, Ling, & Chu, 2017) have all been used as solutions to the issues caused by monoculture soils. Also, in this nutrient‐limited system, fertilization was considered an effective approach to replenish soil nutrients (Zhang et al., 2018). However, the overuse of chemical fertilizers results in severe consequences, such as soil hardening and salinization as well as high levels of pollution in ecosystems (Huang et al., 2018). To combat this, organic fertilizers such as animal manure, straw, animal residues have been applied to reduce the use of agrochemical products (Zhao, Chen, et al., 2016; Zhao, Tian, et al., 2016).

However, the benefits of animal manure, straw, animal residues, rapeseed cake in stimulating plant growth are slow to appear (Zhang et al., 2018). Meanwhile, a variety of microbial species have been reported to promote plant growth and increase plant resistance, include species of *Azospirillum* (Marks et al., 2015), *Enterobacter* (Akkopru & Demir, 2005), *Pseudomonas* (Manikandan, Saravanakumar, Rajendran, Raguchander, & Samiyappan, 2010), *Bacillus* (Maung, Choi, Nam, & Kim, 2017; Shi, Bai, et al., 2017; Shi, Du, et al., 2017), and *Trichoderma* (Singh & Kumar, 2011). Combining these two methods, bio‐organic fertilizers are composed of animal manure, straw, animal residues or rapeseed cake and beneficial bacterial strains. Numerous studies have reported that bio‐organic fertilizers benefit soil quality and soil enzyme activities (Li, Li, et al., 2017; Li, Tao, et al., 2017; Nedunchezhiyan, Byju, Dash, & Ranasingh, 2013), which can stimulate soil microbial biomass and diversity, promote plant growth, and reduce the incidence of FWD (Dicko et al., 2018; Qiu et al., 2012). Conversely, field studies have reported that a single application of bio‐organic fertilizer enhanced with one or two antagonistic microbes only increased the *Fusarium* wilt disease reduction percentage (DRP) to 40%–50%, or the suppressive effect was inconsistent (Shen et al., 2018). These results are not ideal for farmers and are unsustainable in terms of producing high‐yield plants.

Soil fumigants have been used for many years to reduce the incidence of disease and to ensure plant growth because they are versatile, highly effective, and relatively easy to use (Shen et al., 2018). Over the past several decades, methyl bromide was the most commonly used fumigant, until it was banned globally due to its ability to damage the ozone layer (Huang et al., 2018). As the use of methyl bromide and its derivatives continue to be phased out, the need to develop alternative treatment options has arisen. Methyl bromide has been replaced by dazomet, chloropicrin, allyl isothiocyanates, and so on, which are now commonly used worldwide in the production of various plants (Momma, Kobara, Uematsu, Kita, & Shinmura, 2013; Tian, Li, & Sun, 2014). Also noteworthy is the fact that soil fumigation may reduce soil biomass, soil enzyme activity, and the diversity of soil microbial communities, resulting in unstable soil ecosystems that can be easily invaded by soil pathogens (Deng, Parham, Hattey, & Babu, 2006). Therefore, the addition of beneficial microbes after soil fumigation may lead to improved soil biodiversity and health.

Soil microbial activity is an important indicator of soil quality and health, and it plays an important role in soil structure, the nutrient biogeochemical cycle, and ecosystem functioning (Berg & Smalla, 2009; Zhen, Gu, Hu, & Chen, 2018). This activity is sensitive to environmental changes, such as tillage (Sun et al., 2018), fertilization (Xiong et al., 2017), seasonal variation (Legay et al., 2012), and plant type (Wu, Zhao, Hui, & Shao, 2013). Asadishad et al. (2018) and Zaborowska, Woźny, Wyszkowska, and Kucharski (2018) all demonstrated that soil amendments, such as metal nanoparticles and organic fertilizers, affected the soil microbiome in different ways. Additionally, microbial diversity, community composition, and the activities of microorganisms are significant factors in maintaining the sustainability and productivity of ecosystems (Zhang et al., 2018). Therefore, exploring the shifts in the soil microbiome under different management methods and their effects on the environment could help farmers to choose appropriate management strategies that reduce soil disturbance and improve plant growth (Afzaal, Mukhtar, Malik, Murtaza, & Nazar, 2018).

A particular feature of chrysanthemum is that its root system is relatively shallow. As a result, producers seldom disturb the soil below a depth of about 15 cm. Such relatively undisturbed soils often become compacted below this depth, which exacerbates the issues caused by monoculture and the development of FWD. Many studies have concluded that deep tillage is a convenient and effective method for nutrient utilization in the deep soil and soil physical and chemical properties improvement in monoculture‐based production systems (Monsefi, Sharma, & Zan, 2018; Zhai et al., 2017). To the best of our knowledge, little information is available on the effect of deep tillage on chrysanthemum growth. And studies into the practical effects of deep tillage combined with biofertilizer and soil fumigation on shifts in soil structure, diversity, and microbial communities are scarce. The present research set out to address the following questions: (a) How do different soil treatments affect chrysanthemum growth and the incidence of Fusarium wilt disease? (b) How do different soil treatments influence soil microbiomes and soil quality? (c) What are the major environmental factors affecting the composition of soil microbes？

## 2 MATERIALS AND METHODS

### 2.1 Site description and plant materials

The experiments were conducted at the Chrysanthemum Germplasm Resource Preserving Center (Nanjing, China). The field site had been cropped continuously with chrysanthemum for the past seven years, and the soil was heavily infested with *Fusarium oxysporum* f. sp. *chrysanthemi*. The soil pH was 6.96, its specific conductance was 467.7 μS/cm, its organic matter content was 11.6 g/kg, and its levels of available nitrogen, phosphorus, and potassium were 111, 36, and 184 mg/kg, respectively. Before being transplanted to the field, cuttings of the cultivar “*Jinba*,” provided by Honghua Horticulture Co. Ltd. (Shanghai, China), were first established through culturing in perlite for 20 days in a greenhouse with a 16‐hr photoperiod and relative humidity of 70%. The day and night temperatures were maintained at 28°C and 22°C, respectively.

### 2.2 Preparation bio‐organic fertilizer and fumigant

The bio‐organic fertilizer used in the experiment was provided by the Jiangsu Provincial Key Laboratory of organic solid waste utilization. It comprised a 1:1 mixture of processed oil rapeseed cake and pig manure compost. The former was prepared by fermenting oil rapeseed cake at <50°C for 7 days, resulting in a product which consisted of 44.2% organic matter, 12.9% amino acids and oligopeptides, 4.4% nitrogen, 2.3% phosphorus pentoxide, and 0.7% potash. The pig manure compost was purchased from Tianniang Ltd. (Suzhou, China) and was made by composting pig manure at 30–70°C for 25 days. The manure contained 30.4% organic matter, 2.0% nitrogen, 3.7% phosphorus pentoxide, and 1.1% potash. *Paenibacillus polymyxa* (strain SQR21), known to be highly antagonistic to *F. oxysporum* (Fu et al., 2017), was added to the biofertilizer at a rate of ~5.0 × 10^9^ colony‐forming units per g. Dazomet (3,5‐dimethyl‐1,3,5‐thiadiazinane‐2‐thione, ≥95.0% purity) was purchased from Shizhuang Chemical Industry Co. Ltd. (Nantong, Jiangsu, China).

### 2.3 Experimental design

The 15‐plot experiment was set out as a randomized complete block with three replicates and five treatments. Each plot measured 1.6 m × 0.4 m and was planted with 48 rooted cuttings. Before planting, 12 of the plots were plowed to a depth of ~20 cm, and the other three to a depth of ~40 cm. The five treatments were as follows: (1) Control (nontreated), (2) B (1.50 kg bio‐organic fertilizer per m^2^), (3) D (30 g dazomet per m^2^), (4) DB (30 g dazomet plus 1.50 kg bio‐organic fertilizer per m^2^), and (5) DB + 40dp (plowing to a depth of 40 cm plus 30 g dazomet and 1.50 kg bio‐organic fertilizer per m^2^). For treatments (3) through (5), the soil was irrigated to field capacity, after which dazomet microgranules were worked into the upper soil layer. Following this, the soil surface was covered with a plastic film for 20 days before being left exposed for a further seven days before planting.

### 2.4 Plant growth and disease incidence

Shoot height and diameter, shoot dry weight, leaf width and length, root fresh and dry weight, flower diameter, and ray floret number were all measured. For these measurements, twelve plants were sampled randomly from each replicate at flowering time (110 days after transplanting). The wilt symptoms were observed in the field, and pathogens were isolated from sampled plants using PDA culture medium and identified by observation of spore morphology and amplification of the ITS gene region sequence. And the sequences were compared with GenBank using the nucleotide blast software provided online by the National Center for Biotechnology Information. Also, the isolated pathogen was inoculated with chrysanthemum according to Koch's postulates, and its symptoms were consistent with those of the sampled plants. The disease incidence (DI) score for each plot was calculated from the ratio of infected to noninfected plants present, and a disease reduction percentage (DRP) was derived from the following expression: (1 − [DT/DC]) × 100 (Zhao, Chen, et al., 2016; Zhao, Tian, et al., 2016), in which DC (disease incidence of Control) and DT (disease incidence of treatment) were the DI values of the nontreated and treated plots, respectively.

### 2.5 Soil chemical properties and enzymatic activities

Five soil samples were taken from each plot at the depth of 15 cm using a five‐point sampling method at flowering time (110 days after transplanting). All soil samples were sieved through a 2.0‐mm mesh and were thoroughly homogenized. Then, soil samples were divided into two sub‐samples: One was air‐dried at room temperature for seven days to analyze soil chemical properties, and the other was stored at 4℃ to analyze soil enzymatic activity. The amount of available nitrogen was determined using an alkaline hydrolysis diffusion method (Kumar, Dhaliwal, Singh, Lamba, & Ram, 2016), phosphorus content was determined by extraction in NaHCO_3_ (Daroub, Gerakis, Ritchie, Friesen, & Ryan, 2003), and potassium content was determined by extraction in ammonium acetate, followed by flame photometry (Nelson and Heidel, 1952). The organic matter content was determined following the methodology outlined in Ivezic (Ivezić et al., 2016). Catalase activity (expressed as ml 0.1 M KMnO_4_ consumed g^−1^·soil day^−1^) was determined by titration (Achuba & Peretiemoclarke, 2008). Urease activity was determined using a sodium phenolate sodium hypochlorite colorimetric method (Garc A‐Gil, Plaza, Soler‐Rovira, & Polo, 2000). Sucrase activity was determined by 3,5‐dinitrosalicylic acid colorimetry, following the protocol published in Zhao, Chen, et al. (2016) and Zhao, Tian, et al. (2016). Finally, β‐d‐glucosidase activity was determined using a soil β‐d‐glucosidase activity assay kit purchased from Keming Biotechnology Co., Ltd. (Suzhou, Jiangsu, China) as per the manufacturer's protocol.

### 2.6 DNA extraction, PCR amplification, and Illumina sequencing

Three soil samples were taken from each plot at flowering time. Each soil sample was derived from three randomly selected plants. Whole plants were up‐rooted, and all soil that was not tightly adhered to the roots was shaken off; the remaining soil was subjected to the analyses described below. Genomic DNA was extracted from 250 mg samples of rhizosphere soil using a Power Soil DNA Isolation kit (MoBio Laboratories). The concentration and integrity of the resulting DNA were determined using a NanoDrop 2000 UV spectrometer. The DNA was used as a template in PCRs driven by the 515F/806R (Itoh et al., 2014), which targets the V4 hypervariable region of the 16S rRNA gene, and ITS5‐1737F/TS2‐2043R (Zhao et al., 2014), which targets internal transcribed spacer region (ITS2), primer pairs. Amplicons were separated electrophoretically through 2% agarose gels, and those producing strong signals corresponding to a fragment length of 200–300 bp were retained for sequencing. Each pair of amplicons were mixed in equimolar amounts and then purified using a Gel Extraction kit (Qiagen). Sequencing libraries were generated using a TruSeq^®^ DNA PCR‐Free Sample Preparation kit (Illumina), and index codes were added. The quality of the library was assessed using a Qubit 2.0 fluorometer and a Bioanalyzer 2100 device (Agilent Technologies). The library was sequenced using a HiSeq2500 device (Illumina), and 250 bp paired‐end reads were generated by Novogene Biotechnology Inc.

Raw data containing adapters or low‐quality reads would have affected the assembly and analysis. Thus, to obtain high‐quality clean reads, raw reads were further filtered using FASTP (Chen, Zhao, et al., 2018; Chen, Zhou, Chen, & Gu, 2018). Paired‐end clean reads were annotated as raw tags using FLASH (version 1.2.11; Tanja & Salzberg, 2011), and noisy sequences of raw tags were filtered by the QIIME (version 1.9.1; Caporaso et al., 2010) pipeline following the SOP to obtain the high‐quality clean tags. Clean tags were searched against the reference database to perform reference‐based chimera checking using UCHIME algorithm. All chimeric tags were removed, and the resulting effective tags were used for further analysis. The effective tags were clustered into operational taxonomic units (OTUs) of ≥97% similarity using the UPARSE (Edgar, 2013) pipeline. We chose a representative sequence from each OTU, and the Ribosomal Database Project (RDP) classifier (the RDP Bacterial 16S database for 16S rRNA data and the UNITE Fungal ITS database for ITS data) was used to assign taxonomic information. The MOTHUR (version 1.25.1; Zhang et al., 2018) standard operating procedure (SOP) was employed for further analyses.

### 2.7 Statistical analyses and sequence data analyses

Statistical analyses of all parameters were performed using the IBM SPSS statistical software package version 20 (IBM Corporation). Data from each treatment were analyzed using one‐way analysis of variance (ANOVA), and Duncan's multiple range tests (*p* < .05) were performed for multiple comparisons. A principal coordinate analysis (PCoA) was used to visualize the multidimensional data. The PCoA analysis was implemented using routines included in the R v2.15.3 software. Analysis of similarities (ANOSIM) was completed in R v2.15.3. The unweighted pair group method with arithmetic means (UPGMA) clustering was performed to interpret the distance matrices, as implemented in QIIME software. A redundancy analysis (RDA) and a partial RDA were conducted using the “vegan” package in R to assess the effect of the nutrient content and enzyme activity of the soil. Raw bacterial 16S and fungal ITS sequence data are available at the National Center for Biotechnology Information (NCBI) under accession number PRJNA558207.

## 3 RESULTS

### 3.1 Chrysanthemum growth and the incidence of Fusarium wilt disease

A summary of the growth measurements taken from plants at flowering time is given in Table 1. The tallest plants were those grown in the DB + 40dp‐treated soil, followed by plants exposed to the DB, D, and B treatments. Shoot height did not differ significantly between plants grown in the D‐ and DB‐treated soils. The thickest (shoot diameter) and heaviest (shoot dry weight) shoots were generated in plants grown in the DB + 40dp‐treated soil. The widest leaves also developed in plants exposed to this treatment, although the differences among the DB + 40dp‐, DB‐, and D‐treated plants were nonsignificant. Concerning leaf length, there was no apparent effect of treatment. The heaviest (both wet and dry weight) roots were formed by plants grown in the DB + 40dp‐treated soil. Both inflorescence diameter and flower ray floret number were positively affected by each of the treatments, but only significantly increased in plants grown in the DB + 40dp soil.

**TABLE 1:** Effects of soil treatment on chrysanthemum growth parameters at flowering

Treatment	Shoot			Leaf		Root		Flower	
	Height (cm)	Diameter (cm)	Dry wt (g)	Width (cm)	Length (cm)	R. fresh wt (g)	R. dry wt (g)	Diameter (cm)	Ray floret number (No.)
Control	51.50 ± 2.18d	4.00 ± 0.26c	5.63 ± 0.06d	2.23 ± 0.06b	3.53 ± 0.34b	1.01 ± 0.12c	0.25 ± 0.03d	9.03 ± 0.32d	162.00 ± 2.65d
B	61.00 ± 3.50c	4.40 ± 0.20c	7.27 ± 0.09c	2.32 ± 0.21b	3.83 ± 0.29ab	2.06 ± 0.04a	0.46 ± 0.04b	10.40 ± 0.17c	172.36 ± 2.37c
D	68.33 ± 3.79b	4.90 ± 0.05b	9.43 ± 0.44b	2.59 ± 0.22ab	4.41 ± 0.11a	1.42 ± 0.19b	0.28 ± 0.04c	11.45 ± 0.17b	184.33 ± 1.15b
DB	69.56 ± 2.58b	5.02 ± 0.12ab	10.35 ± 0.14b	2.71 ± 0.08a	4.25 ± 0.30a	1.85 ± 0.40ab	0.52 ± 0.02b	12.02 ± 0.46b	183.67 ± 3.21b
DB + 40dp	72.49 ± 3.78a	5.31 ± 0.24a	12.13 ± 0.48a	2.84 ± 0.07a	4.62 ± 0.43a	2.08 ± 0.39a	0.60 ± 0.06a	13.80 ± 0.62a	204.33 ± 4.01a

Note

Different letters indicate significant differences between treatments according to Duncan's multiple range test at the *p* < .05 level. Treatments were as follows: Control; no treatment, B; 1.50 kg bio‐organic fertilizer per m^2^, D; 30 g dazomet per m^2^, DB; combination of D and B, DB + 40dp; combination of DB and tillage to a depth of 40 cm.

**FIGURE 1:**
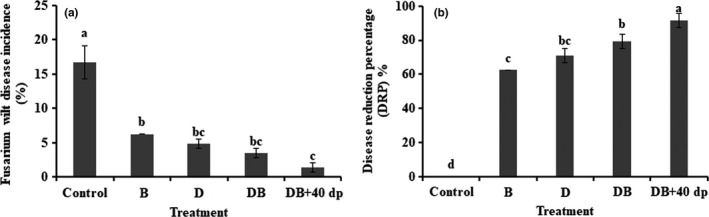
Control of FWD achieved through fungicidal, bio‐organic fertilizer, and tillage methods. (a) disease incidence, (b) disease reduction percentage (DRP). Bars and lines represent mean values of three replicates ±*SE*. A different letter at the head of a column indicates a significant difference (*p* < .05) from other treatments. Treatments were as follows: Control; no treatment, B; 1.50 kg bio‐organic fertilizer per m^2^, D; 30 g dazomet per m^2^, DB; combination of D and B, DB + 40dp; combination of DB and tillage to a depth of 40 cm

All the soil treatments had a suppressive effect on FWD, significantly (*p* < .05) reduced the value of FWD (Figure 1a), and resulted in a significantly (*p* < .05) increment in the DRP (Figure 1b). The highest DI was found in plants grown in the control (nontreated) soil, reaching a cumulative value of 16.7%, compared to values of <6.2% in each of the other treatments. The highest DRP was associated with the DB + 40dp treatment, followed by the DB, D, and B treatments.

### 3.2 Soil properties and enzymatic activities

A summary of the soil properties at flowering time is given in Table 2. Both the B and DB + 40dp soils contained significantly (*p* < .05) more nitrogen and phosphorus than any of the other three treatments (control, D, and DB). The DB + 40dp treatment also contained significantly (*p* < .05) more potassium and organic matter than any of the other treatments. The poorest quality soils in terms of available nitrogen and phosphorus were those in the control and D treatments.

**TABLE 2:** Effects of soil treatment on soil properties at flowering

Treatment	Alkalized N (mg/kg)	Available P (mg/kg)	Available K (mg/kg)	Organic C (g/kg)
Control	92.63 ± 2.14c	3.52 ± 0.33d	64.97 ± 2.08d	2.60 ± 0.86d
B	183.17 ± 2.91a	37.37 ± 0.11a	257.63 ± 1.53b	12.71 ± 0.83b
D	92.17 ± 5.83c	28.50 ± 0.33c	92.63 ± 0.58c	10.99 ± 0.09c
DB	152.37 ± 7.18b	29.82 ± 0.17b	256.97 ± 1.15b	12.71 ± 0.83b
DB + 40dp	187.37 ± 14.09a	37.70 ± 0.42a	266.97 ± 1.15a	14.93 ± 0.96a

Note

Values are means ± standard deviation (*n* = 3). Means followed by the same letter for a given factor are not significantly different (*p* < .05; Duncan test). Treatments were as follows: Control; no treatment, B; 1.50 kg bio‐organic fertilizer per m^2^, D; 30 g dazomet per m^2^, DB; combination of D and B, DB + 40dp; combination of DB and tillage to a depth of 40 cm.

**FIGURE 2:**
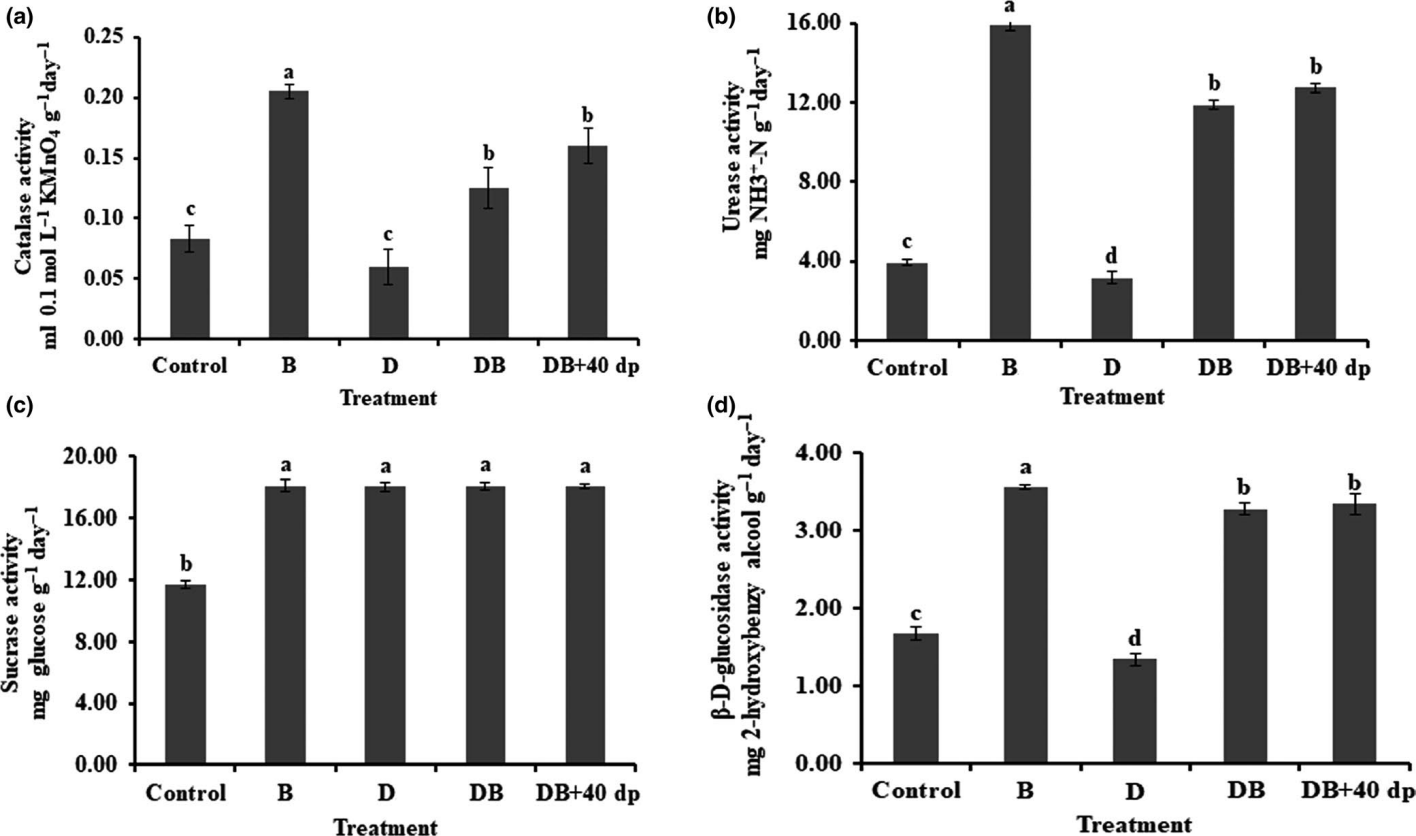
The effects of the various soil treatments on rhizosphere enzyme activities. (a) Catalase, (b) urease, (c) sucrase, (d) β‐d‐glucosidase. Bars and lines represent mean values of three replicates ±*SE*. A different letter at the head of a column indicates a significant difference (*p* < .05) from other treatments. Treatments were as follows: Control; no treatment, B; 1.50 kg bio‐organic fertilizer per m^2^, D; 30 g dazomet per m^2^, DB; combination of D and B, DB + 40dp; combination of DB and tillage to a depth of 40 cm

The activities of catalase (Figure 2a), urease (Figure 2b), and β‐d‐glucosidase (Figure 2d) in the soil were significantly raised in the B treatment. The level of catalase activity was 2.5‐fold higher in the B‐treated soil than in the nontreated soil. The level of activity was 2.0‐fold higher in the DB + 40dp‐treated soil than in the control, was 1.5‐fold higher in the DB‐treated soil, and was 0.75‐fold lower in the D‐treated soil. The highest level of urease activity was found in the B‐treated soil (4.0‐fold higher than the nontreated soil), followed by the DB + 40dp‐treated soil (3.2‐fold) and the DB‐treated soil (3.0‐fold); the level in the D‐treated soil was only 0.8‐fold that of the nontreated soil. The levels of β‐d‐glucosidase activity the B‐, DB + 40dp‐, and DB‐treated soils were 2.1‐, 2.0‐, and 2.0‐fold those of the nontreated soil, respectively, while β‐d‐glucosidase activity in the D‐treated soil was only 0.8‐fold that of the nontreated soil. There was no significant variation in soil sucrase activity among the various treatments, although they all produced higher levels than the nontreated soil (Figure 2c).

### 3.3 Microbiome composition

**FIGURE 3:**
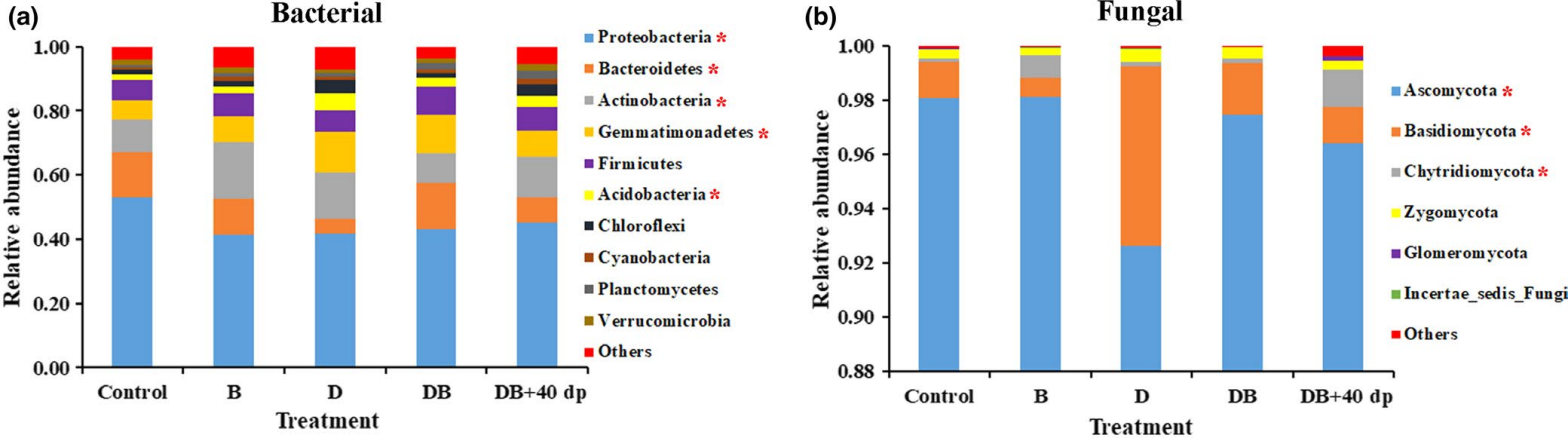
The relative abundance of microbial phyla in the rhizosphere soil as affected by the various soil treatments. (a) bacterial phyla, (b) fungal phyla. “Others” refers to low abundance (<0.5%) phyla. Significant differences between the soil treatments and the control were marked with “*” following the phylum name. Treatments were as follows: Control; no treatment, B; 1.50 kg bio‐organic fertilizer per m^2^, D; 30 g dazomet per m^2^, DB; combination of D and B, DB + 40dp; combination of DB and tillage to a depth of 40 cm

The overall taxonomic complexity of the rhizosphere soil microbiome at the phylum level is presented in Figure 3. The 10 most abundant phyla in rank order were as follows: Proteobacteria, Bacteroidetes, Actinobacteria, Gemmatimonadetes, Firmicutes, Acidobacteria, Chloroflexi, Cyanobacteria, Planctomycetes, and Verrucomicrobia, which together accounted for >93% of the species predicted to be represented based on ribosomal gene sequencing (Figure 3a). The various treatments significantly (*p* < .05) raised the relative abundances of Actinobacteria and Gemmatimonadetes, while they significantly (*p* < .05) suppressed the relative abundances of Proteobacteria and Bacteroidetes. The D treatment had the strongest promotional effect on the abundance of Acidobacteria, whereas the B treatment resulted in the lowest recorded abundance of Bacteroidetes. Ascomycota and Basidiomycota were the two most abundant fungal phyla (Figure 3b). The DB + 40dp treatment favored the growth of Chytridiomycota, while the D treatment promoted the growth of Basidiomycota.

**FIGURE 4:**
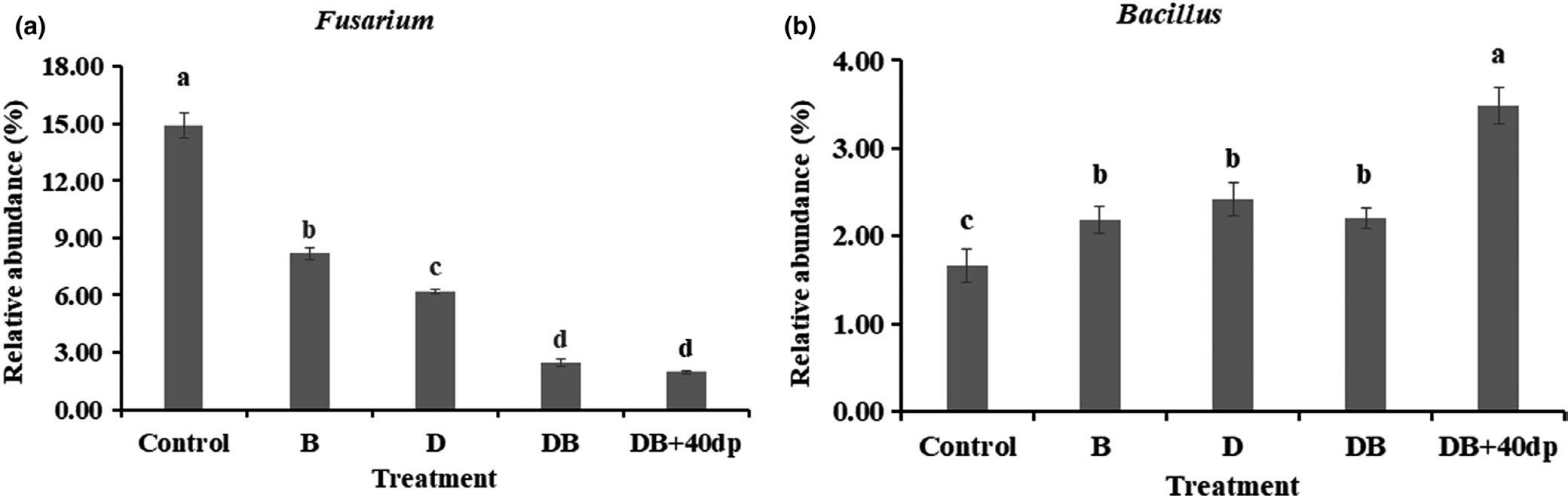
The relative abundance of key microbial genera in the rhizosphere soil as affected by the various soil treatments. (a) *Fusarium* spp., (b) *Bacillus* spp. Bars and lines represent mean values of three replicates ±*SE.* Treatments were as follows: Control; no treatment, B; 1.50 kg bio‐organic fertilizer per m^2^, D; 30 g dazomet per m^2^, DB; combination of D and B, DB + 40dp; combination of DB and tillage to a depth of 40 cm

At the genus level, the five most abundant bacterial taxa were as follows: *Schlegelella*, *Streptomyces*, *Mariniflexile*, *Stenotrophomonas*, and *Bacillus* (Table A1). The various treatments significantly (*p* < .05) promoted the relative abundances of *Schlegelella*, *Streptomyces*, *Stenotrophomonas*, and *Bacillus*, and suppressed the relative abundance of *Mariniflexile* (Table A1). Species belonging to the genera *Alternaria*, *Microidium, Fusarium, Chaetomium,* and *Gymnascella* were the most abundant fungi taxa (Table A2). For the abundance of *Fusarium* spp., the DB + 40dp treatment was the most suppressive (Figure 4a). And the DB + 40dp treatment encouraged the growth of *Bacillus* spp. (Figure 4b).

### 3.4 Microbiome diversity

Bacterial alpha diversity was highest in the DB + 40dp‐treated soil, and lowest in the nontreated soil (Table 3), while fungal diversity was significantly decreased in each of the treatments. Concerning the bacterial components of the microbiome, the DB + 40dp treatment resulted in the highest values for both Chao1 richness and Faith's phylogenetic diversity, and these were significantly (*p* < .05) higher than in the other treatments. In contrast, for the fungal components, the DB + 40dp treatment induced the lowest values for both Chao1 richness and Faith's phylogenetic diversity. The B treatment produced a more even population of fungi, while none of the treatments affected population evenness with respect to the bacterial component. As illustrated in Table A3, there was a positive (*R* = 0.954, *p* = .012) correlation between bacterial alpha diversity (Shannon index) and the root fresh weight of chrysanthemum, while there were significantly negative correlations between fungal alpha diversity (Shannon index) and chrysanthemum growth indices including shoot height, shoot diameter, shoot dry weight, leaf width and length, root fresh weight and dry weight, flower diameter, and a number of flower ray florets.

**TABLE 3:** Alpha diversity indices for the bacterial and fungal components of the rhizosphere soil microbiome, as affected by the soil treatments

Microbe	Treatment	Diversity (Shannon)	Richness (Chao1)	Faith's PD	Evenness
Bacteria	Control	8.97 ± 0.04c	2,895.96 ± 17.48d	233.70 ± 2.32c	0.993 ± 0.001a
	B	9.35 ± 0.02b	4,001.32 ± 17.98b	260.64 ± 2.59b	0.994 ± 0.000a
	D	9.01 ± 0.03c	3,732.79 ± 34.02c	256.89 ± 2.82b	0.993 ± 0.001a
	DB	9.07 ± 0.09c	3,867.58 ± 29.74c	258.82 ± 2.38b	0.993 ± 0.001a
	DB + 40dp	9.57 ± 0.06a	4,445.55 ± 36.77a	295.70 ± 2.88a	0.993 ± 0.004a
Fungi	Control	4.68 ± 0.17a	743.45 ± 17.53a	361.14 ± 18.38a	0.812 ± 0.016b
	B	4.16 ± 0.23b	567.81 ± 14.97b	189.41 ± 2.15b	0.890 ± 0.017a
	D	3.57 ± 0.16c	505.20 ± 15.29c	185.74 ± 2.27b	0.824 ± 0.014b
	DB	3.28 ± 0.22c	500.69 ± 13.41c	159.83 ± 1.97c	0.845 ± 0.017ab
	DB + 40dp	1.94 ± 0.11d	322.59 ± 2.45d	119.06 ± 1.81d	0.881 ± 0.016a

Note

Values are means ± standard deviation (*n* = 3). Means followed by the same letter for a given factor are not significantly different (*p* < .05; Duncan test). Treatments were as follows: Control; no treatment, B; 1.50 kg bio‐organic fertilizer per m^2^, D; 30 g dazomet per m^2^, DB; combination of D and B, DB + 40dp; combination of DB and tillage to a depth of 40 cm.

**FIGURE 5:**
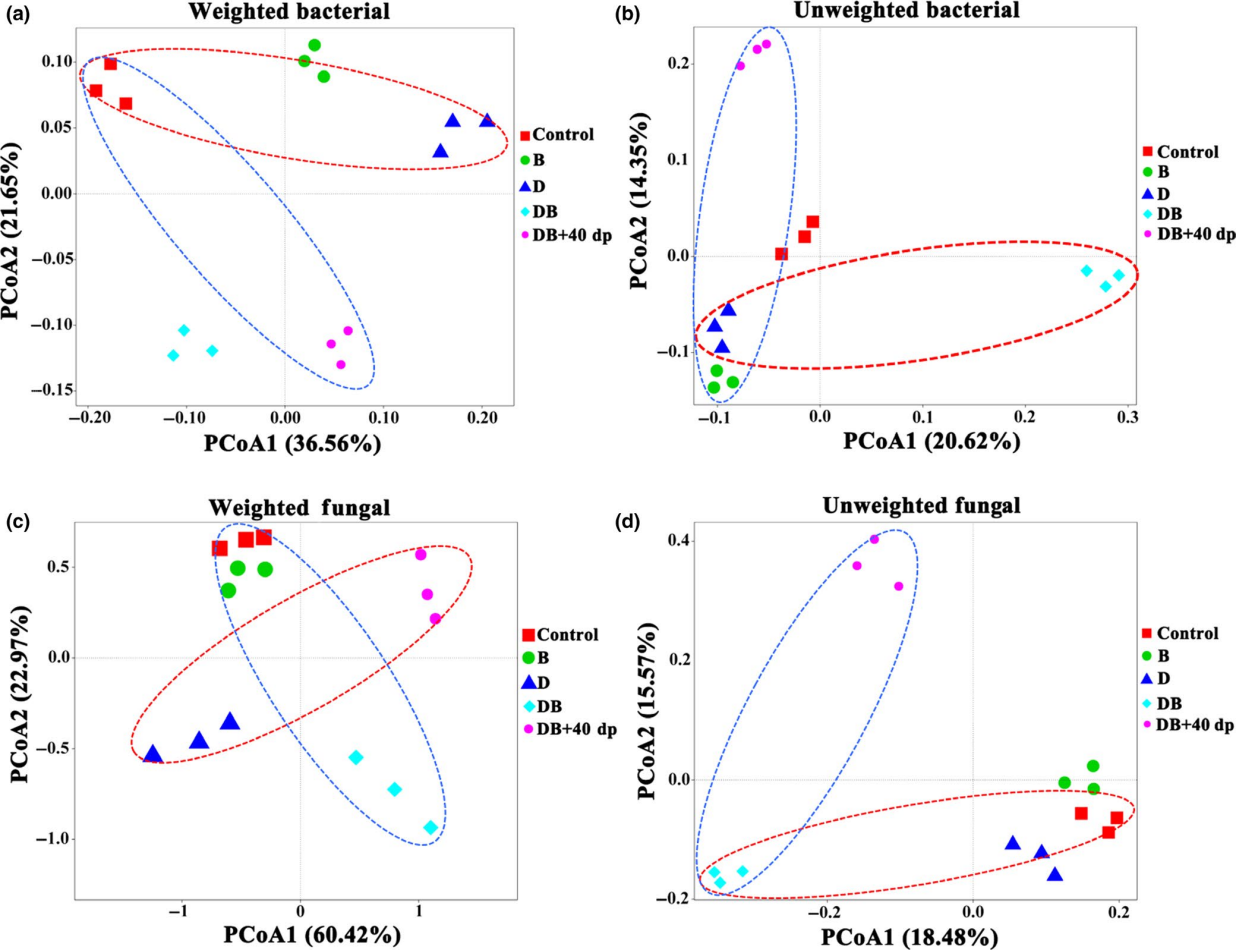
The structure of the rhizosphere soil microbiome as affected by the various soil treatments. (a, c) UniFrac weighted PCoAs of the (a) bacterial and (c) fungal components. (b, d) UniFrac unweighted PCoAs of the (b) bacterial and (d) fungal components. Treatments were as follows: Control; no treatment, B; 1.50 kg bio‐organic fertilizer per m^2^, D; 30 g dazomet per m^2^, DB; combination of D and B, DB + 40dp; combination of DB and tillage to a depth of 40 cm

Following a PCoA, based on either weighted or unweighted UniFrac distances, some of the treatments were found to have generated distinctive soil microbiome compositions. Concerning the bacterial component, the analysis based on the weighted UniFrac distances revealed a separation between the control and D‐treated (ANOSIM, Control vs. D, *p* < .05) soils and the other three soils along with the first principal component (PCoA1), while the control and DB + 40dp‐treated (ANOSIM, Control vs. DB + 40dp, *p* < .05) soils were separated from the other three soils along PCoA2 (Figure 5a). The analysis based on the unweighted UniFrac distances revealed that the D‐ and DB‐treated (ANOSIM, D vs. DB, *p* < .05) soils were distinct from the other three soil along PCoA1, and the B‐ and DB + 40dp‐treated (ANOSIM, B vs. DB + 40dp, *p* < .05) soils differed from the other treatments along PCoA2 (Figure 5b). For the fungal component, the analysis based on weighted UniFrac distances revealed that the D‐ and DB + 40dp‐treated (ANOSIM, D vs. DB + 40dp, *p* < .05) soils were separated from the other three soils along PCoA1, while the nontreated and DB‐treated (ANOSIM, Control vs. DB, *p* < .05) soils were distinct from the other three soils along PCoA2 (Figure 5c). Based on unweighted UniFrac distances, the analysis revealed that the D‐ and DB‐treated (ANOSIM, D vs. DB, *p* < .05) soils differed from the other three soils along PCoA1, while the DB‐ and DB + 40dp‐treated (ANOSIM, DB vs. DB + 40dp, *p* < .05) soils were separated from the other three along PCoA2 (Figure 5d).

**FIGURE 6:**
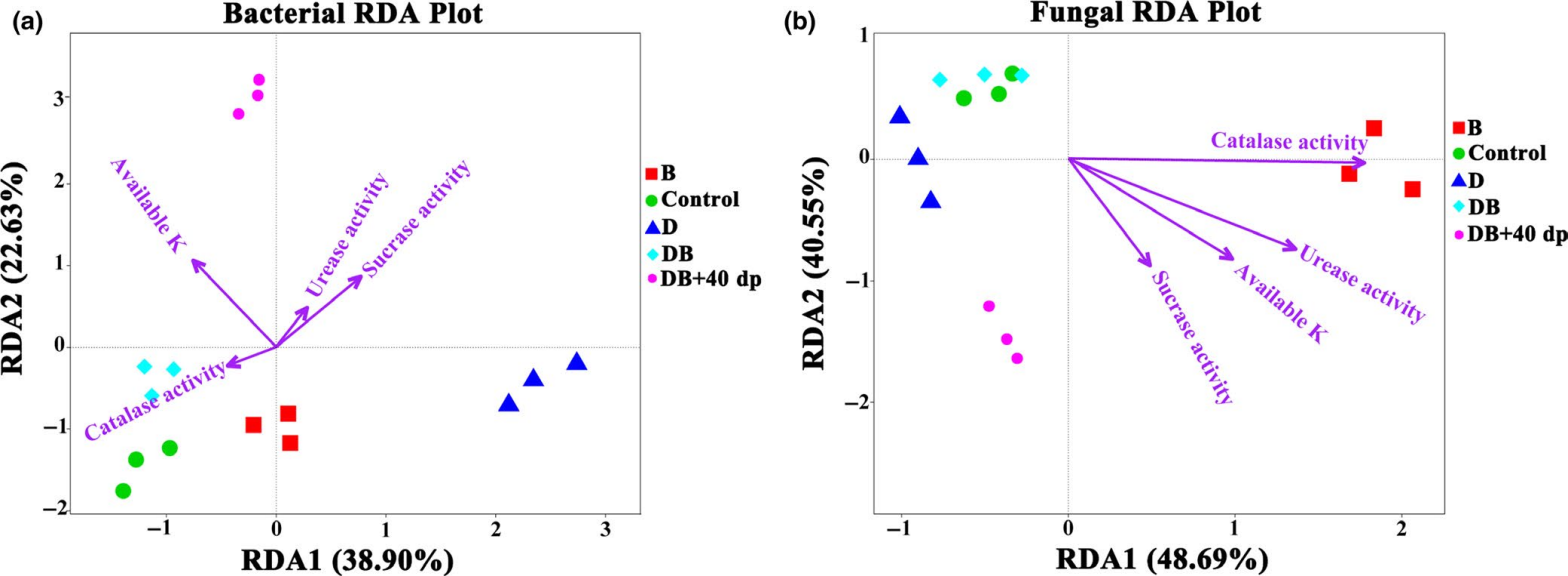
The influence of key environmental factors on the composition of the rhizosphere soil microbiome. The RDAs refer to (a) the bacterial and (b) the fungal components. The length of each arrow indicates the contribution of the parameter to the structural variation. Treatments were as follows: Control; no treatment, B; 1.50 kg bio‐organic fertilizer per m^2^, D; 30 g dazomet per m^2^, DB; combination of D and B, DB + 40dp; combination of DB and tillage to a depth of 40 cm

Finally, an RDA analysis implied that the microbiome composition was influenced by potassium availability and enzymatic activity in the rhizosphere soil. The first two axes explained 38.9% and 22.6% of the variability related to the bacterial component (Figure 6a), respectively, and 48.7% and 40.6% of the variability in the fungal component (Figure 6b), respectively. Concerning the variability in both the bacterial and the fungal components of the microbiome, the availability of potassium and the activities of catalase, urease, and sucrase were all significant factors.

## 4 DISCUSSION

The most significant finding of the present study is that combining deep tillage with mild dazomet fumigation and dressing with a bio‐organic fertilizer encouraged chrysanthemum growth better than using either dazomet fumigation or a bio‐organic fertilizer alone. Meanwhile, each of the treatments suppressed FWD to some extent, and the DB + 40dp treatment was the most effective. It has previously been shown that supplementing fertilizers with spores of an antagonistic microbe such as *Bacillus subtilis* or *P. polymyxa* is beneficial in terms of promoting growth (Suliasih & Widawati, 2018), controlling FWD (Huang et al., 2019), and raising yield (Schütz et al., 2017). Indirani, Jayakumar, and Latha (2000) reported that dazomet fumigation improved the growth, yield, and quality of tomato and Mark and Cassells (1999) found that dazomet fumigation enhanced the strawberry productivity. Also, soil tillage has been shown to increase the effectiveness of organic matter application at increasing nutrient accumulation and crop yield (Shi, Bai, et al., 2017; Shi, Du, et al., 2017).

The extent of the enzyme activity in the soil is an important indicator of soil health (Dick, Pankhurst, Doube, & Gupta, 1997), as a number of the reactions catalyzed by these enzymes contribute to the availability of plant nutrients and the neutralization of toxic elements (Marcinkevicinen, Boguzas, Balnyte, Pupaliene, & Velicka, 2013). Here, the highest levels of soil catalase, urease, and β‐d‐glucosidase activity were recorded in the B‐treated soil, in agreement with the results of related studies (Li, Li, et al., 2017; Li, Tao, et al., 2017; Zhao, Tian, et al., 2016). In contrast, enzyme activity is not only compromised as a result of dazomet fumigation (Zhao, Tian, et al., 2016) but also declined with years of repeated fumigation applications (Chen, Zhao, et al., 2018; Chen, Zhou, et al., 2018). This decline in enzyme activity is likely due to the release of several microbial inhibitors, notably methyl isothiocyanate, formaldehyde, and hydrogen sulfide (Stromberger, Klose, Ajwa, Trout, & Fennimore, 2005). A decline in nutritional status probably also exerts a detrimental influence on the microbiome structure of the rhizosphere soil (Pathan et al., 2015).

The rhizosphere microbiome contributes significantly to the health and productivity of plants (Bakker, Chaparro, Manter, & Vivanco, 2015). Each of the treatments resulted in increases in both the diversity and richness of the bacterial component of the microbiome and had the opposite effect on the fungal component. This effect was particularly marked in the DB + 40dp treatment, which implies that increasing the depth of soil tillage is likely beneficial where chrysanthemum is grown as a monocrop; specifically, this intervention should support crop productivity by altering the composition and spatial distribution of nutrients and the microbiome (Sun et al., 2018), resulting in promoting the root exudates of plants (Zhu, Vivanco, & Manter, 2016). The achieved improvements in soil conditions can be expected to encourage the development of populations of microbial antagonists of FWD such as *P. polymyxa* (Shi, Bai, et al., 2017; Shi, Du, et al., 2017). Various soil treatments have been associated with significant effects on the species composition of the soil microbiome across a range of agro‐ecosystems (Shen et al., 2014). The abundance of Proteobacteria species has been reported to be positively correlated with carbon availability (Cleveland, Nemergut, Schmidt, & Townsend, 2007), while the abundance of Actinobacteria species is frequently associated with disease suppression (Trivedi et al., 2017; Xiong et al., 2017). Here, the abundance of Proteobacteria was reduced in each of the treatments (Figure 3a), while the abundance of Actinobacteria was enhanced. This was especially true for the B‐treated soil. Members of the Ascomycota and Basidiomycota phyla dominated the fungal component of the rhizosphere soil microbiome, consistent with observations made in soils supporting peanut, pea, vanilla crops and so on (Li, Ding, Zhang, & Wang, 2014; Xiong et al., 2017; Xu, Ravnskov, Larsen, Nilsson, & Nicolaisen, 2012). The Ascomycota phylum, which accounted for >92% of the fungal component of the rhizosphere soil microbiome across all treatments, also contains many plant pathogens (Li et al., 2016). This group tends to be suppressed in soils where the disease is controlled (Shen et al., 2015). Here, a significantly lower abundance of Ascomycota phylum species was noted in each of the treated soils, especially in the D‐ and DB + 40dp‐treated soils. The implication is that dazomet is effective against pathogenic microbes, particularly to *Fusarium* spp. (Nico, 2012). The relative abundances of *Fusarium* spp. were decreased by 45.06% as a result of the B treatment, by 58.36% as a result of the D treatment, by 83.68% as a result of the DB treatment, and by 86.90% as a result of the DB + 40dp treatment.

The relative abundances of species belonging to the *Pseudomonas* (Proteobacteria) and *Bacillus* (Firmicutes) genera were significantly increased in each of treated soils; this was especially the case for the DB + 40dp treatment, in which the abundance of *Pseudomonas* spp. was 1.3‐fold (Table A1) higher than in the nontreated soil, and the abundance of *Bacillus* spp. was 6.4‐fold (Table A1) higher. Species belonging to these two genera are antagonistic toward various plant pathogens, through forming biofilms, inducing systemic resistance, promoting plant growth, and enhancing siderophore production (Ma, Cao, et al., 2017; Ma, Hu, Wang, Xia, & Du, 2017; Ru et al., 2012). It has been reported that certain *Pseudomonas* spp., following colonization of the roots of tomato plants, can secrete acylated homoserine lactones into the rhizosphere, which are important for quorum sensing and pathogen resistance (Chowdhury et al., 2013). It has also been claimed that a strain of *Bacillus* sp. can exert a measure of control over FWD in tomato (Abdallah, Mokni‐tlili, Nefzi, Jabnoun‐khiareddine, & Daami‐remadi, 2016).

## 5 CONCLUSIONS

Overall, these experiments have demonstrated that each of the B, D, DB, and DB + 40dp treatments promoted chrysanthemum growth, provided substantial control over FWD, and altered the composition of the rhizosphere soil microbiome, especially the DB + 40dp treatment. The strong control effect of the DB + 40dp treatment was probably achieved through enhancing the availability of plant nutrients and through promoting the presence of bacteria belonging to the genera *Pseudomonas* (Degrassi et al., 2002), *Bacillus* (Ma, Cao, et al., 2017; Ma, Hu, et al., 2017), *Stenotrophomonas* (Jeong et al., 2010)*,* and of fungi belonging to the genus *Chaetomium* (Shanthiyaa et al., 2013) in the rhizosphere, which all can act as plant growth‐promoting rhizomicrobes. The results suggest that the DB + 40dp treatment is a better control strategy than those presently employed by commercial chrysanthemum producers.

## CONFLICTS OF INTEREST

None declared.

## AUTHOR CONTRIBUTION

Huijie Chen: Conceptualization (equal); Data curation (equal); Formal analysis (equal); Writing‐original draft (equal); Writing‐review & editing (equal). Shuang Zhao: Conceptualization (equal); Funding acquisition (equal); Project administration (equal); Writing‐review & editing (equal). Jiamiao Zhao: Methodology (equal); Software (equal). Kaikai Zhang: Methodology (equal); Resources (equal); Software (equal). Jing Jiang: Methodology (equal); Software (equal). Zhiyong Guan: Funding acquisition (equal); Project administration (equal); Supervision (equal). Sumei Chen: Funding acquisition (equal); Project administration (equal); Supervision (equal). Fadi Chen: Funding acquisition (equal); Project administration (equal); Supervision (equal). Weimin Fang: Conceptualization (equal); Funding acquisition (equal); Project administration (equal). 

## ETHICS STATEMENT

None required.

## Data Availability

Raw bacterial 16S and fungal ITS sequence data are available at the National Center for Biotechnology Information (NCBI) under accession number PRJNA558207: https://www.ncbi.nlm.nih.gov/bioproject/PRJNA558207. Other data are provided in the results section of this paper.

## APPENDIX 1

**TABLE A1:** Effects of the soil treatments on the abundance of bacterial genera in the rhizosphere soil

Genus	Control	B	D	DB	DB + 40dp
*Schlegelella*	0.07 ± 0.01a	0.10 ± 0.01a	0.09 ± 0.01a	0.05 ± 0.04a	0.38 ± 3.18a
*Streptomyces*	1.79 ± 0.39b	5.25 ± 0.44a	1.95 ± 0.25b	0.96 ± 0.37b	1.29 ± 0.40b
*Mariniflexile*	4.17 ± 0.65a	0.03 ± 0.01b	0.03 ± 0.01b	0.88 ± 0.14b	0.05 ± 0.00b
*Stenotrophomonas*	0.06 ± 0.01a	0.29 ± 0.12a	0.22 ± 0.09a	0.25 ± 0.21a	1.54 ± 1.41a
*Bacillus*	1.66 ± 0.15c	2.18 ± 0.19b	2.41 ± 0.23b	2.20 ± 0.12b	3.49 ± 0.60a
*unidentified_Cellvibrionaceae*	2.93 ± 0.33a	0.09 ± 0.01b	0.12 ± 0.03b	0.12 ± 0.01b	0.28 ± 0.09b
*unidentified_Gemmatimonadaceae*	0.48 ± 0.05c	1.42 ± 0.07b	2.86 ± 0.44a	0.49 ± 0.03c	0.43 ± 0.03c
*Lactococcus*	1.69 ± 0.26a	1.42 ± 0.21a	1.21 ± 0.18a	1.99 ± 0.19a	2.16 ± 0.60a
*Sphingomonas*	1.08 ± 0.03bc	1.27 ± 0.06b	2.93 ± 0.23a	0.93 ± 0.17bc	0.75 ± 0.04c
*Chitinophaga*	0.98 ± 0.06b	2.44 ± 0.18a	0.82 ± 0.15b	0.80 ± 0.13b	0.23 ± 0.04c
*Polycyclovorans*	0.27 ± 0.01b	0.14 ± 0.02b	0.25 ± 0.02b	0.32 ± 0.02b	2.15 ± 0.34a
*Steroidobacter*	1.02 ± 0.21b	1.10 ± 0.15b	1.94 ± 0.31a	0.69 ± 0.12b	1.22 ± 0.17b
*Glycomyces*	1.02 ± 0.40ab	1.77 ± 0.38a	0.29 ± 0.05b	0.63 ± 0.29b	0.28 ± 0.12b
*Methylobacillus*	1.07 ± 0.05bc	1.04 ± 0.18bc	0.59 ± 0.09c	2.02 ± 0.29a	1.16 ± 0.09b
*unidentified_Thaumarchaeota*	0.07 ± 0.04b	1.56 ± 0.46a	0.03 ± 0.01b	0.09 ± 0.03b	0.08 ± 0.03b
*Devosia*	2.05 ± 0.12a	0.76 ± 0.06c	0.42 ± 0.10c	1.60 ± 0.17b	1.53 ± 0.12c
*Pseudomonas*	0.36 ± 0.02c	1.44 ± 0.16b	0.46 ± 0.01c	1.68 ± 0.19b	2.32 ± 0.15a
*Gemmatimonas*	0.20 ± 0.01d	1.03 ± 0.08b	1.53 ± 0.10a	0.46 ± 0.03c	0.56 ± 0.06c
*Nonomuraea*	0.19 ± 0.03b	1.24 ± 0.29a	0.47 ± 0.12b	0.34 ± 0.22b	0.56 ± 0.22b
*H16*	0.35 ± 0.03bc	0.40 ± 0.03bc	1.24 ± 0.18a	0.18 ± 0.01c	0.54 ± 0.08b
*Gaiella*	0.34 ± 0.03cd	0.54 ± 0.04b	1.50 ± 0.06a	0.20 ± 0.03d	0.41 ± 0.08bc
*Escherichia‐Shigella*	0.02 ± 0.01a	0.36 ± 0.12a	0.64 ± 0.46a	0.11 ± 0.08a	0.02 ± 0.01a
*Acidibacter*	0.41 ± 0.10bc	0.44 ± 0.04bc	1.06 ± 0.22a	0.17 ± 0.03c	0.63 ± 0.11b
*Aureimonas*	1.20 ± 0.08a	0.35 ± 0.04bc	0.08 ± 0.02d	0.50 ± 0.04b	0.27 ± 0.03c
*Actinomadura*	0.10 ± 0.02b	0.78 ± 0.27a	0.11 ± 0.01b	0.07 ± 0.01b	0.10 ± 0.02b
*Haliangium*	0.27 ± 0.02c	0.52 ± 0.04bc	0.96 ± 0.13a	0.71 ± 0.14ab	0.74 ± 0.05ab
*Ramlibacter*	0.13 ± 0.01c	0.11 ± 0.03c	0.11 ± 0.03c	1.07 ± 0.08a	0.51 ± 0.04b
*Pseudolabrys*	0.59 ± 0.06b	0.92 ± 0.06a	1.03 ± 0.08a	1.06 ± 0.10a	0.40 ± 0.05b

Note

Values are means ± standard deviation (*n* = 3). Different letters indicate significant differences (*p* < .05) among soil treatments. Treatments were as follows: Control; no treatment, B; 1.50 kg bio‐organic fertilizer per m^2^, D; 30 g dazomet per m^2^, DB; combination of D and B, DB + 40dp; combination of DB and tillage to a depth of 40 cm.

**TABLE A2:** Effects of the soil treatments on the abundance of fungal genera in the rhizosphere soil

Genus	Control	B	D	DB	DB + 40dp
*Alternaria*	1.78 ± 0.88a	0.53 ± 0.18a	20.71 ± 5.65a	42.31 ± 22.19a	29.08 ± 19.58a
*Microidium*	42.09 ± 4.75a	51.26 ± 10.50a	54.09 ± 12.47a	11.23 ± 9.58b	9.27 ± 3.08b
*Fusarium*	14.89 ± 0.67a	8.18 ± 0.30b	6.20 ± 0.10c	2.43 ± 0.19d	1.95 ± 0.08d
*Chaetomium*	0.38 ± 0.03a	0.17 ± 0.04a	0.03 ± 0.01a	0.19 ± 0.08a	2.39 ± 2.07a
*Gymnascella*	0.33 ± 0.08b	0.02 ± 0.00b	3.47 ± 1.41a	0.02 ± 0.00b	0.17 ± 0.03b
*Arthrobotrys*	0.01 ± 0.00b	0.00 ± 0.00b	0.01 ± 0.00b	0.00 ± 0.00b	4.16 ± 1.49a
*Cladosporium*	0.54 ± 0.08b	0.61 ± 0.08b	0.31 ± 0.10b	2.29 ± 1.05a	2.27 ± 0.43a
*Paraphoma*	1.99 ± 0.75a	3.00 ± 0.30a	0.03 ± 0.01b	0.01 ± 0.00b	0.21 ± 0.06b
*Phialosimplex*	0.05 ± 0.03b	0.01 ± 0.00b	2.48 ± 0.66a	0.01 ± 0.00b	0.05 ± 0.02b
*Trichoderma*	0.15 ± 0.02a	1.01 ± 0.83a	0.02 ± 0.01a	0.02 ± 0.01a	0.03 ± 0.00a
*Talaromyces*	0.04 ± 0.02b	1.62 ± 0.57a	0.09 ± 0.03b	0.04 ± 0.01b	0.01 ± 0.00b
*Emericellopsis*	0.50 ± 0.11b	0.09 ± 0.02b	0.18 ± 0.04b	0.12 ± 0.04b	1.36 ± 0.53a
*Conocybe*	1.12 ± 0.62a	0.00 ± 0.00b	0.01 ± 0.01b	0.00 ± 0.00b	0.01 ± 0.00b
*Ustilago*	1.21 ± 0.56a	0.26 ± 0.02b	0.30 ± 0.18b	0.06 ± 0.03b	0.12 ± 0.09b
*Monographella*	0.49 ± 0.27ab	1.23 ± 0.56a	0.02 ± 0.01b	0.21 ± 0.14b	0.01 ± 0.00b
*Xanthoria*	0.71 ± 0.58a	0.07 ± 0.01a	0.07 ± 0.02a	0.04 ± 0.02a	0.29 ± 0.08a
*Madurella*	0.00 ± 0.00b	0.01 ± 0.01b	0.00 ± 0.00b	0.69 ± 0.40a	0.03 ± 0.01b
*Ilyonectria*	0.84 ± 0.19a	0.10 ± 0.02b	0.01 ± 0.01b	0.02 ± 0.00b	0.56 ± 0.23a
*Pyrenula*	0.00 ± 0.00b	0.00 ± 0.00b	0.70 ± 0.21a	0.01 ± 0.01b	0.09 ± 0.02b
*Catenaria*	0.00 ± 0.00a	0.00 ± 0.00a	0.00 ± 0.00a	0.00 ± 0.00a	0.42 ± 0.32a
*Monoblepharis*	0.02 ± 0.00b	0.00 ± 0.00b	0.03 ± 0.01b	0.04 ± 0.01b	0.47 ± 0.28a
*Acremonium*	0.67 ± 0.15a	0.35 ± 0.21ab	0.32 ± 0.23ab	0.09 ± 0.01b	0.23 ± 0.07ab
*Gonapodya*	0.02 ± 0.00b	0.00 ± 0.00b	0.39 ± 0.20a	0.01 ± 0.01b	0.05 ± 0.02b
*Cercophora*	0.43 ± 0.13a	0.04 ± 0.02a	0.01 ± 0.00a	0.29 ± 0.14a	0.30 ± 0.22a
*Geosmithia*	0.07 ± 0.03b	0.57 ± 0.12a	0.00 ± 0.00b	0.02 ± 0.01b	0.03 ± 0.01b
*Rhodosporidium*	0.01 ± 0.00b	0.01 ± 0.00b	0.01 ± 0.00b	0.01 ± 0.00b	0.31 ± 0.18a
*Pachykytospora*	0.08 ± 0.06a	0.23 ± 0.18a	0.00 ± 0.00a	0.00 ± 0.00a	0.00 ± 0.00a
*Calostilbe*	0.26 ± 0.09a	0.08 ± 0.02b	0.01 ± 0.00b	0.10 ± 0.04b	0.03 ± 0.01b
*Remispora*	0.04 ± 0.01b	0.02 ± 0.02b	0.00 ± 0.00b	0.19 ± 0.11a	0.02 ± 0.01b

Note

Values are means ± standard deviation (*n* = 3). Different letters indicate significant differences (*p* < .05) among soil treatments. Treatments were as follows: Control; no treatment, B; 1.50 kg bio‐organic fertilizer per m^2^, D; 30 g dazomet per m^2^, DB; combination of D and B, DB + 40dp; combination of DB and tillage to a depth of 40 cm.

**TABLE A3:** Correlation analysis of microbe diversity index and growth index

Growth index	Diversity of bacteria			Diversity of fungus		
	*R*	*p*	Sig.	*R*	*p*	Sig.
Shoot height	0.435	.464		−0.906	.034	*
Shoot diameter	0.489	.403		−0.938	.018	*
Shoot dry wt	0.655	.23		−0.991	.001	**
Leaf width	0.443	.455		−0.946	.015	*
Leaf length	0.451	.446		−0.913	.031	*
Root fresh wt	0.954	.012	*	−0.97	.006	**
Root dry wt	0.498	.394		−0.955	.011	*
Flower diameter	0.517	.372		−0.825	.085	
Flower ray floret	0.66	.225		−0.99	.001	**

Note

Treatments were as follows: Control; no treatment, B; 1.50 kg bio‐organic fertilizer per m^2^, D; 30 g dazomet per m^2^, DB; combination of D and B, DB + 40dp; combination of DB and tillage to a depth of 40 cm.
